# Identification of potential bioactive phytochemicals for the inhibition of platelet-derived growth factor receptor β: a structure-based approach for cancer therapy

**DOI:** 10.3389/fmolb.2024.1492847

**Published:** 2024-10-15

**Authors:** Insan Habib, Md Nayab Sulaimani, Deeba Shamim Jairajpuri, Afzal Hussain, Taj Mohammad, Mohamed F. Alajmi, Anas Shamsi, Md Imtaiyaz Hassan

**Affiliations:** ^1^ Department of Parasitology, Faculty of Science, University of South Bohemia, Ceske Budejovice, Czechia; ^2^ Centre for Interdisciplinary Research in Basic Sciences, New Delhi, India; ^3^ Department of Medical Biochemistry, College of Medicine and Health Sciences, Arabian Gulf University, Manama, Bahrain; ^4^ Department of Pharmacognosy, College of Pharmacy, King Saud University, Riyadh, Saudi Arabia; ^5^ Centre of Medical and Bio-Allied Health Sciences Research, Ajman University, Ajman, United Arab Emirates

**Keywords:** platelet-derived growth factor receptor beta, phytochemicals, virtual screening, molecular dynamics simulation, essential dynamics

## Abstract

Platelet-derived growth factor receptor beta (PDGFRβ) belongs to the receptor tyrosine kinase (RTK) protein family and is implicated in several disorders such as hematopoietic, glial, and soft-tissue cancer, non-cancerous disorders, including skeletal defects, brain calcification, and vascular anomalies. The research on small molecule inhibitors targeting PDGFRβ in cancer treatment has seen promising developments, but significant gaps remain. PDGFRβ, receptor tyrosine kinase, is overexpressed in various cancers and plays an important role in tumor progression, making it a potential therapeutic target. However, despite advances in identifying and characterizing PDGFRβ inhibitors, few have progressed to clinical trials, and the mechanistic details of PDGFRβ′s interactions with small molecule inhibitors are still not fully understood. Moreover, the specificity and selectivity of these inhibitors remain challenging, as off-target effects can lead to unwanted toxicity. In this investigation, two compounds, Genostrychnine and Chelidonine, were discovered that help inhibit the kinase activity of PDGFRβ. These small molecules were identified by employing various parameters involved in the drug discovery process, such as Lipinski’s rule of five (RO5), 2D similarity search and 3D pharmacophore-based virtual screening followed by MD simulation studies. The identified molecules were found to be effective and significantly bound with the PDGFRβ kinase domain. Overall, our findings demonstrate that these small drug-like compounds can be beneficial tools in studying the properties of PDGFRβ and can play a crucial role in the therapeutic development of cancers and other associated diseases.

## Introduction

Receptor tyrosine kinases (RTKs) are a class of transmembrane proteins that function as receptors for cytokines, growth factors, hormones, and other signaling agents (Fredriksson et al., 2004; Roskoski Jr, 2018). Within the human body, there exist 58 recognized RTKs that share a comparable protein structure. The RTKs are composed of distinct structural components. Their structure includes an extracellular domain responsible for binding with ligands, a transmembrane helix that anchors the receptor in the cell membrane, and an intracellular region housing both tyrosine kinase domain and c-terminal tail (Robinson et al., 2000; Manning et al., 2002; Du and Lovly, 2018). Each RTK has a receptor-specific binding site and a ligand binding site that can induce its dimerization. The catalytic domain lies in the c-terminal region with the most significant level of conservation; these domains are responsible for the receptor’s kinase activity (Lemmon and Schlessinger, 2010). RTKs play a role in tissue segmentation, organogenesis, and maintaining adult organismal homeostasis by promoting communication between cells and their extracellular environment (Lemmon and Schlessinger, 2010; Trenker and Jura, 2020).

The platelet-derived growth factor receptor (PDGFR) is a member of the RTK family that comes in two isoforms: α and β, each encoded by the *PDGFRA* and *PDGFRΒ* genes, respectively (Fredriksson et al., 2004; Roskoski Jr, 2018). The physiological roles of both the PDGFRs have been inferred to embryonic development, and PDGFRβ is mainly implicated in blood vessel formation and kidney development as shown in mice models (Andrae et al., 2008; Demoulin and Essaghir, 2014). Additionally, new functions such as bone regeneration, heart regeneration, and adipose tissue homeostasis were asserted to PDGFRβ in the latest mouse studies (Böhm et al., 2019; Yue et al., 2019). PDGFRβ is expressed within the hematopoietic system, specifically in erythroid and myeloid precursors located in the bone marrow. Moreover, it is also found in mature megakaryocytes, fibroblasts, epithelial cells, osteoblasts, and monocytes, indicating its involvement in monocytic differentiation (Yoon et al., 2000; Steer and Cross, 2002). Its expression in smooth muscle cells plays a role in vascular repair. The receptor mediates monocyte, macrophage, and platelet involvement in inflamed tissue, promoting inflammation and regulating tissue interstitial fluid pressure (Heldin et al., 1998; Heuchel et al., 1999; Steer and Cross, 2002).

Structurally, PDGFRβ, which comprises 1106 amino acids, is organized into distinct domains: an extracellular ligand-binding domain, a single transmembrane helix, and an intracellular tyrosine kinase domain. The extracellular region contains five immunoglobin-like (Ig-like) domains (33aa-524aa) that are essential for binding PDGF ligands. The intracellular region includes a juxta membrane domain, a split tyrosine kinase domain (600aa-962aa) with an ATP-binding site, and a regulatory C-terminal tail. Specifically, the active site residue Asp826 is located within the kinase domain and is pivotal for the receptor’s kinase activity. The correct folding and interaction of these domains are critical for PDGFRβ′s function in signal transduction pathways, which are often dysregulated in various diseases, including cancers and vascular disorders (Fredriksson et al., 2004; Chen et al., 2013).

The elevated expression of PDGFRβ is linked with various diseases, including cancer. Additionally, aberrant PDGFRβ signaling and its activation have been implicated in human meningioma, where PDGFRβ is constitutively activated, contributing to tumor growth and proliferation (Shamah et al., 1997); whereas, in atherosclerosis, PDGFRβ plays a crucial role in the proliferation and migration of vascular smooth muscle cells and neointimal formation which is a key process in the development of atherosclerosis (Sirois et al., 1997). A recent study found that PDGFRβ signaling promotes high mobility group box 1 (HMGB1) expression in mechanically stretched vascular muscle cells, which leads to vascular difficulties (Kim et al., 2022). The phosphorylation activity of PDGFRβ was significantly active in the mechanically stretched (MS) muscle cells, suggesting the involvement of the receptor kinase in MS-muscle cells. Interestingly, in PDGFRβ deficient cells, MS-induced HMGB1 secretion was significantly decreased (Heldin and Lennartsson, 2013; Kim et al., 2022). Most solid tumors express PDGFRβ on endothelial or perivascular cells (Pietras et al., 2003a; Lewis, 2007), and the interstitial pressure in solid tumors impedes the passage of cytotoxic chemotherapy (Lewis, 2007).

PDGFRβ signaling has been reported to increase interstitial pressure within tumors, whereas inhibiting phosphorylation activity reverses the effect (Pietras et al., 2001). Moreover, the critical role of PDGFRβ in regulating interstitial fluid pressure becomes evident with the frequent edema seen in patients under PDGFR inhibitor treatment, such as imatinib. Additionally, imatinib’s ability to slow growth in children with chronic myeloid leukemia implies that PDGF receptors are involved in bone formation processes post-birth (Millot et al., 2014; Guérit et al., 2021). When PDGF receptors were coupled with antagonists, the antitumor effectiveness of chemotherapeutics was found to be enhanced (Pietras et al., 2002; Pietras et al., 2003b). Imatinib, i.e., the first tyrosine kinase inhibitor (TKI), sorafenib, and axitinib are oral agents that inhibit the PDGFRβ activity (Druker et al., 2001). Dasatinib, an oral TKI, reduces PDGF tyrosine kinase activity and receptor activation by PDGF, 67-fold more potentially than imatinib (Chen et al., 2006). Sunitinib has both anti-tumor and anti-angiogenic effects against PDGF receptors. It is a multitargeted receptor TKI and shows high potential in inhibiting RTKs (Mendel et al., 2003).

According to earlier research, imatinib inhibits PDGFRβ kinase. Gastrointestinal stromal tumors (GIST) can be treated with imatinib with a remarkable degree of success (Azribi et al., 2009). Therefore, further exploration of the structural biology of PDGFRβ holds great potential in facilitating the development of novel therapeutic interventions (Manley et al., 2002). Nevertheless, more selective molecules targeting PDGFRβ with improved efficacy are required for effective therapeutic. Phytochemicals are one of the major sources of therapeutics, which have an essential role in the pharmaceutical industry (Shakya, 2016; Trinh et al., 2020; Anjum et al., 2022b). IMPPAT stands as one of the most extensive curated databases focusing on phytochemicals, specifically designed for virtual screening purposes (Mohanraj et al., 2018; Yang et al., 2022).

In this research, we employed a multitier screening strategy to evaluate the collection of phytochemical compounds derived from the IMPPAT database, incorporating Lipinski’s rule of five (RO5). RO5 is a set of guidelines used to evaluate the drug-likeness of a chemical compound, particularly its oral bioavailability. It is based on observing that most orally active drugs have certain molecular properties in common. The rule helps in predicting whether a compound is likely to be an effective oral drug based on its pharmacokinetic profile. A compound is more likely to have good oral bioavailability if its molecular weight is less than 500 Da, LogP is less than 5, the number of hydrogen bond donors are not more than 5, and the number of hydrogen bond accepter are not more than 10. Initially, we identified the top candidates by analyzing their binding modes and affinity scores. Subsequently, we eliminated compounds lacking PAINS patterns using the SwissADME server. Additionally, we leveraged the pkCSM server to assess the ADMET properties of the selected compounds (Pires et al., 2015). Finally, compounds that passed the used assessments and were bonded specifically towards the binding site of PDGFRβ were selected, and all-atom molecular dynamics (MD) simulation was performed to estimate the conformational dynamics and stability of elucidated compounds.

## Materials and methods

### Bioinformatics resources

This research was carried out on a high-end dual-booted workstation running Windows 10 and Ubuntu 2020 beta. We employed the InstaDock tool for molecular docking. (Mohammad et al., 2021), the Discovery Studio Visualizer, and PyMOL (Lill and Danielson, 2011) For interaction analysis and visualization. IMPPAT (Mohanraj et al., 2018), pkCSM, SwissADME (Daina et al., 2017), PASS (Lagunin et al., 2000), and other web-based servers and resources were used for different tasks of the study.

### Receptor and library preparation

The human PDGFRβ kinase domain structure (including amino acids L600-L962) was modelled through the Phyre2 server (Kelley et al., 2015), and refined in InstaDock. The final structure was energy minimized in PyPAN (https://hassanlab.org/pypan/) and assigned with the appropriate atom types. The refined model was then evaluated through the PROCHECK server (Laskowski et al., 1993) and Ramachandran plots were generated. The screening was carried out on phytochemicals available from the IMPPAT database (Mohanraj et al., 2018). After applying the Lipinski filter, compounds possessing suitable physicochemical properties were extracted from the IMPPAT database. Initially consisting of 9596 compounds, the database was narrowed down to approximately 5800 compounds that met the Lipinski filter criteria.

### Molecular docking screening

Molecular docking screening is considered one of the most valuable methods in drug design and discovery (Jairajpuri et al., 2020; Anjum et al., 2021). In structure-guided drug discovery it enables researchers to predict and assess the interactions between small molecules (ligands) and target proteins (receptors) at the molecular level (Amir et al., 2020; Mohammad et al., 2020a). Here, in this study, to perform molecular docking, we used the InstaDock tool with blind search space, and the grid sizes for X, Y, and Z coordinates were set to 70 Å, 80 Å, and 80 Å, respectively. The centre of the grid was selected for the axes X: 13.29, Y: 0.40, and Z: 0.90 with a grid spacing of 1 Å. The QuickVina-W scoring function was used for the docking calculations, embedded in InstaDock v1.2. The maximum number of docked conformations during the docking run was set to nine. The docking was flexible for the ligands, and the rest of the parameters were set to their default values. The main aim of using InstaDock is to calculate the binding affinity of the phytoconstituents towards PDGFRβ.

### ADMET properties

After identifying potent PDGFRβ binding partners through molecular docking, we evaluated the selected compounds’ ADMET properties using SwissADME and pkCSM. To ensure specificity in our drug design and discovery process, we implemented the PAINS filter. This filter helps to eliminate compounds that display structural patterns known as Pan Assay Interference Compounds (PAINS), which tend to bind to multiple targets non-specifically. PAINS patterns refer to specific structural motifs found in certain chemical compounds that are known to cause false positives in the high-throughput screening (HTS) process. These compounds are notorious for interfering with biological assays through non-specific binding rather than through specific interactions with a biological target. For further analysis, we prioritized compounds with favorable ADMET and drug-like characteristics while excluding those with toxic patterns (Mohammad et al., 2019; Mohammad et al., 2020b; Shafie et al., 2021).

### PASS analysis

The PASS server method represents a valuable tool for assessing the biological properties of chemical compounds through a thorough examination of their chemical structures (Lagunin et al., 2000). This method provides valuable insights into the potential biological activities of compounds, aiding researchers in understanding their pharmacological potential and facilitating the drug discovery process. To evaluate the biological activities of compounds that were filtered through the ADMET criteria, the PASS server was utilized (Lagunin et al., 2000). This approach allows the server to provide valuable insights into the specific biological properties of the compounds under investigation.

### Interaction analysis

PyMOL and Discovery Studio Visualizer were used to examine the compound’s detailed interactions with PDGFRβ that were selected from the PASS analysis. The docked compound’s output files were taken from InstaDock output. PyMOL was used to generate the ribbon representation and the electrostatic potential surface. Within 3.5 Å, hydrogen bonds were mapped and labelled using dotted lines. Discovery Studio Visualizer was used to create two-dimensional plots of the interactions between the compounds and PDGFRβ.

### MD simulation

MD simulation plays a critical role in studying the atomic motions at the protein-ligand interface under solvent conditions (Gonçalves et al., 2017; Gonçalves et al., 2022). By employing MD simulation, we can model and analyze the dynamic behavior of the complex system over time. We performed an MD simulation to support the docking results obtained from the interaction between PDGFRβ and the phytochemicals (Genostrychnine and Chelidonine). PDGFRβ and its docked complexes with Genostrychnine and Chelidonine were simulated using GROMACS v5.5.1 and employed GROMOS 54A7 force fields to determine their structural coordinates (Abraham et al., 2015). We used the ATB server to create the topologies of the ligands. A 10Å distance was placed for each system in the cubic box of an initial dimension of 8 nm to the edges before solvation. The systems were solvated in a virtual box using the SPC216 water model. To neutralize the systems, an appropriate number of counter ions (Na^+^ and Cl^−^) was added. The solvated system’s energy consumption and possible steric hindrance between the atoms were reduced by the steepest descent algorithm by 10,000 steps followed by conjugate gradient methods. Subsequently, each system underwent a gradual heating process from 0 K to 300 K and equilibrated for 100 ps at constant volume and a constant pressure of 1 atm. Finally, a 100 ns production run was performed on each system at constant temperature and pressure. The GROMACS tools were used to investigate the protein-ligand stability from the resulting trajectories. The generated outputs were analyzed for RMSF, RMSD, *R*g, H-bonds, SASA, distance cross-correlation matrix, secondary structure analysis, and PCA (Adnan et al., 2022a; Adnan et al., 2022b; Anjum et al., 2022a; Hassan et al., 2022).

### Principal component analysis and essential dynamics

Various life science measuring approaches collect data for many more variables per sample than the normal number of samples analyzed (Naqvi et al., 2018). PCA is a mathematical approach that decreases the data dimensionality while maintaining the majority of the variation in the data set (Jolliffe, 1982). This is performed by determining the paths along which the data variation is greatest, known as principal components (PCs). The sample can then be plotted, allowing for a more detailed assessment of sample similarities and differences, as well as determining whether the sample can be grouped. Furthermore, PCA reveals high-amplitude motion in the simulated trajectories. Through PCA, we investigated the MD trajectories of the PDGFRβ, PDGFRβ-Genostrychnine, and PDGFRβ-Chelidonine complexes for conformational sampling and stability in PCA and FEL analyses (Anjum et al., 2022b).

### MMPBSA calculation

MMPBSA (Molecular mechanics/Poisson-Boltzmann surface area) is one of the most widely used approaches for estimating the binding free energy of a protein-ligand complex. A short MD trajectory of 10 ns (from 35 to 45 ns) was extracted from the stable region of the PDGFRβ-Genostrychnine, PDGFRβ-Chelidonine, and PDGFRβ-Sunitinib (control) complexes for MMPBSA calculations. The binding energy components were calculated while using the MMPBSA approach of the gmx_mmpbsa package. The gmx_mmpbsa tool uses the following equation to calculate the binding energy of the protein-ligand complex-

$$\Delta G_{\text{Binding}}=G_{\text{Complex}}-\left(G_{\text{Protein}}+G_{\text{Ligand}}\right)$$

where, *G*_Complex_ signifies the total free energy of the binding complex, and *G*_Protein_ and *G*_Ligand_ are the measure of total free energies of PDGFRβ and the compounds Genostrychnine, Chelidonine, and Sunitinib, respectively.

## Results

### Structure modeling

The PDGFRβ kinase domain structure (including amino acids L600-L962) was energy minimized in PyPAN (https://hassanlab.org/pypan/) and assigned with the appropriate atom-types. The Ramachandran plots before and after minimization of the protein structure showed no significant difference and no critical residues in the outlier regions (Supplementary Figure S1). The refined model was then evaluated through the PROCHECK server and superimposed with the AlphaFold predicted model and its neighbouring template of PDGFRα (PDB ID: 5GRN). It showed identical topology with high similarity in structure superimposition with an RMSD of 0.365 Å and 0.436 Å with the AlphaFold and PDGFRα models, respectively (Supplementary Figure S2).

### Molecular docking

Initially, we applied Lipinski’s rule of five (RO5) to select phytochemicals from the IMPPAT database. Approximately 5800 compounds were chosen for molecular docking analysis against PDGFRβ to determine their binding affinities. Subsequently, compounds were filtered based on their binding affinity with PDGFRβ after the docking process. From this screening, we identified the top 30 phytochemical hits with a binding affinity of ≤ −9.5 kcal/mol with PDGFRβ. Table 1 shows the binding affinity of the selected phytochemicals along with two control molecules.

**TABLE 1:** The binding affinity of the top 30 phytochemicals and two reference compounds with PDGFRβ.

S. No.	Phytochemical ID	Phytochemical name	Binding free energy (kcal/mol)	pKi
1.	24901683	4-pyridone analogue 34	−10.4	7.63
2.	164710	Anolobine	−10.3	7.55
3.	104860	Altertoxin I	−10.3	7.55
4.	119204	Roemerine	−10.1	7.41
5.	12313196	(S)-Neolitsine	−10.0	7.33
6.	13462	3-Nitrofluoranthene	−9.9	7.26
7.	197810	Chelidonine	−9.9	7.26
8.	9798203	Balsaminone A	−9.9	7.26
9.	10184	Benzo [a]fluorenone	−9.8	7.19
10.	73393	Genostrychnine	−9.8	7.19
11.	9817839	Dehydroevodiamine	−9.8	7.19
12.	3175−84−6	Norushinsunine	−9.8	7.19
13.	10378981	Michelalbine	−9.8	7.19
14.	101651627	emenolone	−9.8	7.19
15.	11438278	Cryptodorine	−9.7	7.11
16.	344234	Benzo [c]phenanthridine	−9.7	7.11
17.	4970	Protopine	−9.7	7.11
18.	85976174	Lasioerin	−9.7	7.11
19.	94577	Cepharadione A	−9.7	7.11
20.	41679-82-7	Dehydroanonaine	−9.6	7.04
21.	5359405	Bionet1_001411	−9.6	7.04
22.	6453733	N-acetylanonaine	−9.6	7.04
23.	191752	Norlaureline	−9.6	7.04
24.	6005	Apomorphine	−9.6	7.04
25.	124069	Dihydrosanguinarine	−9.5	6.97
26.	443716	Hydroxysanguinarine	−9.5	6.97
27.	5315739	N-Acetyldehydroanonaine	−9.5	6.97
28.	72322	Coptisine	−9.5	6.97
29.	172169	68492-68-2	−9.5	6.97
30.	363863	Maackiain	−9.5	6.97
31.	5291	Imatinib (control)	−9.1	6.67
32.	5329102	Sunitinib (control)	−7.3	5.35

### ADMET properties

ADMET (absorption, distribution, metabolism, excretion, and toxicity) is a set of pharmacokinetic characteristics of a drug candidate. These properties are crucial in evaluating whether a compound is suitable as a drug for human use, as they affect how the drug behaves in the body*.* ADMET analysis assesses the pharmacokinetic properties and potential toxicity of candidate compounds and holds immense significance in the drug discovery process, ensuring the safety of the compounds in the drug development process (Ferreira and Andricopulo, 2019). We conducted a computational analysis of the selected compounds to assess their ADMET properties. During the ADMET analysis (http://www.swissadme.ch/), we also conducted a comprehensive assessment of the compounds’ toxicity to identify any potential adverse effects they might induce. Finally, four compounds exhibited favorable ADMET properties, demonstrating promising characteristics in terms of absorption, distribution, metabolism, excretion, and no toxicity compared to the toxic control molecules (Table 2). Importantly, they were found to be devoid of any PAINS (Pan Assay Interference Compounds) patterns.

**TABLE 2:** ADMET parameters of the identified hits.

Phytochemical name/ID	Absorption		Distribution	Metabolism	Excretion	Toxicity
	GI Absorption	Water solubility	BBB Permeation	CYP2D6 substrate/Inhibitor	OCT2 Substrate	AMEStoxicity
Genostrychnine (73393)	93.904	−2.25	0.023	No	Yes	No
Chelidonine (197810)	91.984	−2.191	−0.228	No	No	No
4-pyridone analogue 34 (24901683)	93.351	−3.731	−1.486	No	No	Yes
(S)-Neolitsine (12313196)	95.567	−3.752	0.116	No	No	Yes
Imatinib	94.3	−3.49	−1.22	No	No	Yes
Sunitinib	90.14	−3.75	−0.9	No	No	Yes

### PASS analysis

The biological properties of phytochemicals must be explored to ensure their effectiveness with the desired characteristics (Jairajpuri et al., 2020). Therefore, we have analyzed the PASS (http://www.way2drug.com/passonline/) of the selected phytochemicals to investigate their predicted biological activity; PASS states that compounds with *P*_*a*_ *> P*_*i*_ are considered as desired ones to show specific biological activity. The *P*_*a*_ (probability “Active”) value indicates the probability that the compound will exhibit a specific biological activity. In contrast, *P*_*i*_ (probability “Inactive”) indicates the probability of the compound not showing any specific activity. According to the PASS analysis, three compounds, Genostrychnine, Chelidonine, and 4-pyridone analogue 34, showed favorable biological properties and can act as antineoplastic alkaloids, platelet adhesion inhibitors, phosphatase inhibitors, and apoptosis agonists, which suggests that the elucidated compounds may possess great potential in anticancer activities and inhibiting PDGFR kinase activity (Table 3). From here onwards, compound (S)-Neolitsine was eliminated from the study due to its irrelevant biological properties.

**TABLE 3:** Predicted biological activities of the chosen compounds.

S. No.	Phytochemical name	*P*_*a*_	*P*_*i*_	Biological activity
1.	Genostrychnine	0.592	0.051	Phosphatase inhibitor
		0.374	0.019	Antineoplastic alkaloid
		0.883	0.005	Respiratory analeptic
2.	Chelidonine	0.500	0.006	Antineoplastic alkaloid
		0.448	0.114	Platelet adhesion inhibitor
		0.346	0.100	Apoptosis agonist
3.	4-pyridone analogue 34	0.687	0.006	Angiogenesis inhibitor
		0.621	0.041	Antineoplastic
		0.551	0.021	PDGFR kinase inhibitor
4.	(S)-Neolitsine	0.949	0.001	Antiparkinsonian, rigidity relieving
		0.891	0.002	Neurotransmitter uptake inhibitor
		0.874	0.006	Antineurotic
5.	Imatinib	0.805	0.005	Protein kinase inhibitor
		0.697	0.003	Growth factor agonist
		0.696	0.002	Janus tyrosine kinase 3 inhibitor
6.	Sunitinib	0.902	0.004	Vascular endothelial growth factor antagonist
		0.860	0.003	PDGFR kinase inhibitor
		0.841	0.005	Angiogenesis inhibitor

### Interaction analysis

**FIGURE 1:**
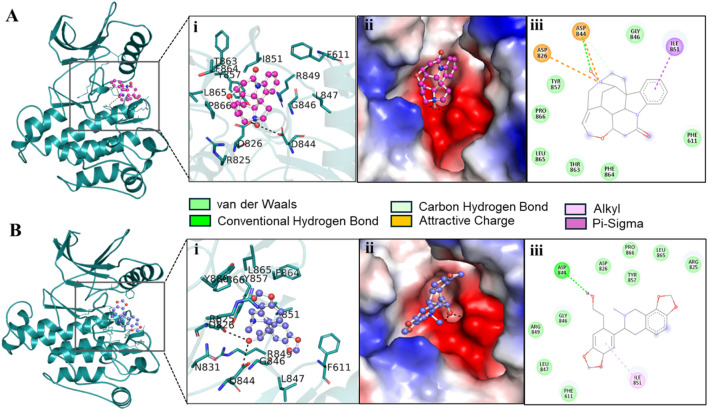
Interactions of **(A)** PDGFRβ with Genostrychnine (pink) and **(B)** Chelidonine (blue). (I) A magnified view of protein-ligand interactions is illustrated in a cartoon. (II) A surface view of PDGFRβ bound with the identified compounds as electrostatic potential. (III) PDGFRβ interactions are represented as 2D diagrams.

We extracted potential docked conformations from the docked-out files of the three compounds, Genostrychnine, Chelidonine, and 4-pyridone analogue 34, when docked with PDGFRβ. Interaction analysis revealed that out of the three molecules, Genostrychnine and Chelidonine showed specific binding with the active site of PDGFRβ, specifically the Asp826 residue, as depicted in Figure 1. Here, in interaction analysis, compound 4-pyridone analogue 34 was not found to interact with the active site residue specifically; hence, it was eliminated from the study due to its non-specific interaction. The figure illustrates the close interaction between Genostrychnine and Chelidonine with the Asp826 (active site), and Phe611, Asp144, and Asp844 (ATP binding sites) residues of PDGFRβ kinase (Figures 1A,B). Additionally, the figure demonstrates the strong complementarity between Genostrychnine and Chelidonine within the deep binding pocket of PDGFRβ [Figures 1A, B (II)].

To get detail insights into how Genostrychnine and Chelidonine interact with the binding site residues, their interactions were further investigated using Discovery Studio Visualizer. The 2D interaction plots showed that Genostrychnine interacts with various close interactions with the PDGFRβ binding site, including the active site residue, Asp826 [Figure 1A (III)]. It showed an attractive charge with Asp826 and Asp844 along with two hydrogen bonds with Asp844, one pi-sigma bond with Ile851, and seven van der Waals interactions with different residues. At the same time, Chelidonine also interacts with various close interactions with the PDGFRβ binding site, including the active site residue, Asp826 [Figure 1B (III)]. It showed one conventional hydrogen bond with Asp844 and one alkyl bond with Ile851 along with nine van der Waals interactions with different residues of the PDGFRβ binding site. These interactions involve conventional hydrogen bonds and attractive charge interactions, highlighting Asp826, Phe611, Asp144, and Asp844 as crucial regions for the functional activity of the kinase. Hence, it could be said that Genostrychnine and Chelidonine could act as potential inhibitors of PDGFRβ.

## MD simulations

To explore the structural behavior and dynamics of protein-ligand complexes, we performed detailed all-atom MD simulations lasting 100 ns for three systems: PDGFRβ-Genostrychnine, PDGFRβ-Chelidonine, and PDGFRβ in its unbound state. During these simulations, we meticulously analyzed various systematic and structural parameters to gain valuable insights into the stability and dynamics of the PDGFRβ-ligand complexes. The findings from these simulations are further discussed in detail.

### Structural dynamics and compactness

**FIGURE 2:**
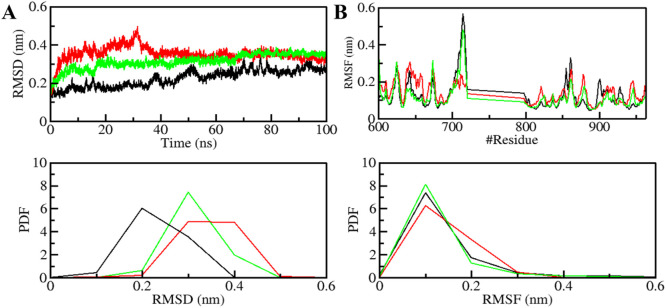
Structural dynamics of PDGFRβ upon Genostrychnine and Chelidonine binding. **(A)** RMSD plot of PDGFRβ complex with Genostrychnine and Chelidonine. **(B)** RMSF plot of the PDGFRβ and its complex with Genostrychnine and Chelidonine. The lower panels depict the probability distribution function (PDF) of the values, with the position of the residues indicated by the symbol “#”.

The structural dynamics of PDGFRβ were assessed by utilizing the root-mean-square deviation (RMSD), a widely used parameter for evaluating the divergence of protein structures over time. The RMSD values for PDGFRβ, PDGFRβ-Genostrychnine, and PDGFRβ-Chelidonine were determined to be 0.22 nm, 0.35 nm, and 0.31 nm, respectively (Table 4). The simulations indicated that the binding of Genostrychnine and Chelidonine to PDGFRβ reached equilibrium and exhibited good stability, as evidenced by the RMSD graph (Figure 2A). In the case of the PDGFRβ-Genostrychnine complex, a slight fluctuation was observed prior to the 30 ns mark, after which the system remained stable and balanced throughout the remainder of the simulation. Conversely, the PDGFRβ-Chelidonine complex demonstrated initial stabilization followed by random fluctuations between 0 and 20 ns. Notably, over the entire 100 ns simulation, all systems exhibited balanced RMSD values, with a slight variation observed in the PDGFRβ-Genostrychnine complex without any significant shifts. Additionally, a probability distribution function (PDF) was plotted to illustrate the distribution of values and their associated probabilities (Figure 2A, lower panel).

**TABLE 4:** The average values of various parameters.

System	RMSD (nm)	RMSF (nm)	*R*_g_ (nm)	SASA (nm^2^)	# H-bonds
PDGFRβ	0.22	0.12	1.97	150	182
PDGFRβ-Genostrychnine	0.35	0.12	1.93	166	195
PDGFRβ-Chelidonine	0.31	0.11	1.95	151	199

^#^Represent number.

Root mean square fluctuation (RMSF) has proven to be a useful method for quantifying the residual vibrations of a protein structure during an MD simulation (Mohammad et al., 2020a; Gupta et al., 2022). It offers insights into how ligand binding influences the residual changes in a protein. The RMSF plot depicted the dynamic behavior of residues in PDGFRβ both before and after the binding of compounds (Figure 2B). The RMSF fluctuation was reduced and stabilized upon Chelidonine binding; moreover, the protein-ligand system was remarkably stable. However, the residual vibration patterns upon Genostrychnine binding were not uniform; in some regions, a slight increase in residual vibrations was observed, while in others, a decrease was noted, potentially indicating the presence of flexible loop regions. In contrast, when comparing the RMSF values, the PDGFRβ-Chelidonine complex exhibited greater stability than the PDGFRβ-Genostrychnine complex. The PDF indicated increased fluctuations in the PDGFRβ-Chelidonine complex, although no significant changes were found compared to the PDGFRβ-Genostrychnine complex (Figure 2B, lower panel).

**FIGURE 3:**
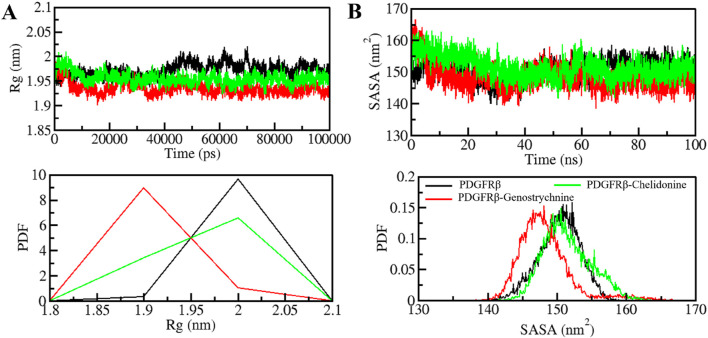
Folding and structural compactness of PDGFRβ upon Genostrychnine and Chelidonine binding. **(A)** *R*_*g*_ plot and **(B)** SASA plot of PDGFRβ with the selected compounds.

The radius of gyration (*Rg*) is closely linked to the folding and compactness of the protein structure and is a valuable factor for studying how compactly a protein is packed in its 3D form (Yadav et al., 2020). Therefore, during the simulation, we studied the *Rg* in time-evolution settings to evaluate the compactness of the PDGFRβ-Genostrychnine and PDGFRβ-Chelidonine complexes (Figure 3A). For the PDGFRβ-Genostrychnine complex, the plot indicated a slight decrease in the *Rg*, whereas the *Rg* value of PDGFRβ-Chelidonine was well along with that of apo PDGFRβ, as also shown in the PDF analysis (Figure 3A, lower panel).

The solvent-accessible surface area (SASA) helps in studying the stability and folding behavior of the protein (Richmond, 1984). During the entire simulation, no change was observed in the SASA value for the PDGFRβ-Chelidonine complex in contrast to the apo PDGF receptor, while the PDGFRβ-Genostrychnine complex was slightly disturbed initially (Figure 3B). Moreover, the PDF analysis showed a minor decrease in the average SASA for the Genostrychnine binding with PDGFRβ (Figure 3B, lower panel).

### Hydrogen bonding

**FIGURE 4:**
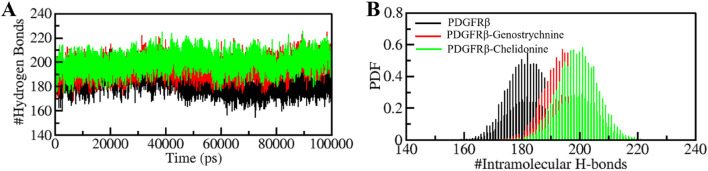
Hydrogen bond analysis. **(A)** Time evolution of intramolecular H-bonds in PDGFRβ. **(B)** PDF of the hydrogen bond distribution. # represents number.

The formation and disruption of hydrogen bonds (H-bonds) are pivotal factors that significantly influence the conformational dynamics of proteins (Hubbard and Haider, 2010). H-bonds play a vital role in protein folding dynamics. To gain insights into the intramolecular bonding of both the compound and PDGFRβ, we conducted an analysis of the time evolution of H-bonds. This analysis allowed us to study the formation and breaking of H-bonds over time, shedding light on the stability and interactions within the protein-ligand complexes. The plot indicated a slight increase in H-bonding in PDGFRβ in a complex with Genostrychnine and Chelidonine. The PDGFRβ before ligand binding exhibited 182 average H-bonds, which increased to 195 and 199 after binding with Genostrychnine and Chelidonine, respectively (Figure 4A). The calculated PDF for the three systems showed good consistency (Figure 4B). As determined from the plots, a slight change in intramolecular H-bonds was noted in the Genostrychnine and Chelidonine complexes in contrast to the free PDGFRβ.

**FIGURE 5:**
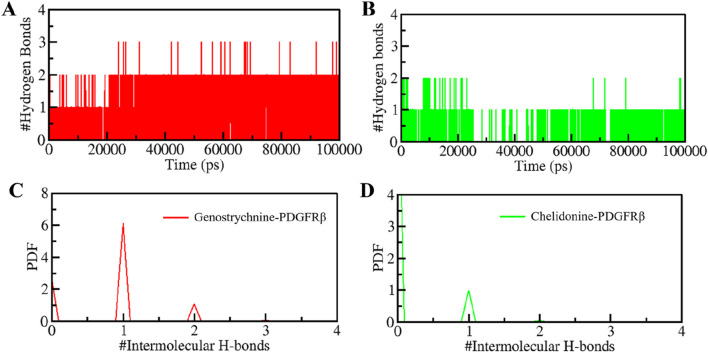
Intermolecular H-bonding between PDGFRβ and **(A)** Genostrychnine (red) and **(B)** Chelidonine (green). **(C, D)** The panels show the PDF of the intermolecular hydrogen bond distribution in the PDGFRβ-ligand complexes. #represents a number.

H-bonds play a significant role in stabilizing protein-ligand complexes (Williams and Ladbury, 2003). H-bonds, along with other noncovalent interactions, should always be considered when designing ligands for target proteins. We evaluated the time evolution of intermolecular H-bonds to ascertain the firmness of H-bonding between the compounds and PDGFRβ. The average number of H-bonds formed in the PDGFRβ-Genostrychnine and PDGFRβ-Chelidonine complex was likely to be one (Figures 5A,B). Furthermore, the PDF suggested uniform intramolecular H-bonds in the PDGFRβ-Genostrychnine system with an average of one, whereas in the PDGFRβ-Chelidonine complex, the H-bonding formed in phases during the entire simulation (Figures 5C,D). Therefore, it can be suggested that the Genostrycnine bonding with the apo PDGFRβ was more stable than the Chelidonine complex and indicated that their initial docking position was unchanged. Moreover, the post-MD simulations snapshots of the docked complexes showed stable interactions where most interactions are preserved (Supplementary Figure S3).

### PCA and FELs analysis

**FIGURE 6:**
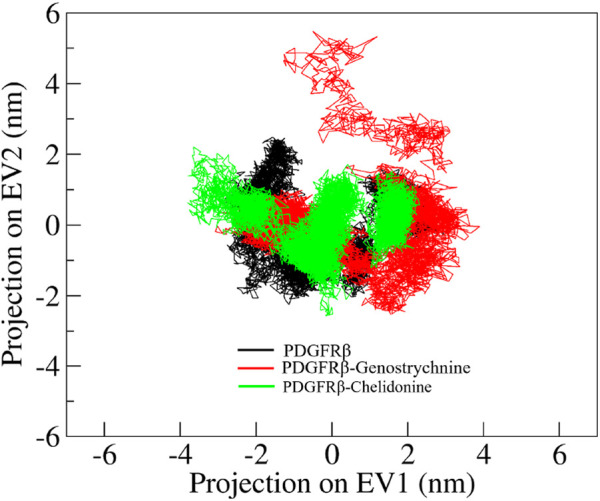
Conformational projections of PDGFRβ, PDGFRβ-Genostrychnine and PDGFRβ-Chelidonine.

Principal component analysis (PCA) is a powerful technique that helps identify the most significant modes of motion and capture the essential conformational changes in the protein and its ligand interactions. We performed PCA to explore the conformational sampling of apo PDGFRβ and its complexes with Genostrychnine and Chelidonine (Figure 6). As shown in the graph, the conformations of PDGFRβ on two different eigenvalues are projected by its *C*α atoms. The plot suggested that the projections of the complexes, PDGFRβ-Genostrychnine and PDGFRβ-Chelidonine, covered the free PDGFRβ clusters. Nevertheless, it was observed that both the complexes had occupied a slightly distinct conformational space. It can be observed that the PDGFRβ-complexes are stable in their conformational space.

**FIGURE 7:**
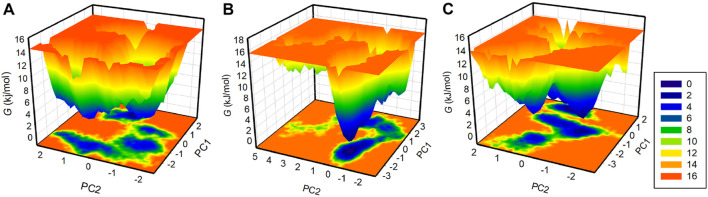
The free energy landscapes for **(A)** PDGFRβ, **(B)** PDGFRβ-Genostrychnine, and **(C)** PDGFRβ-Chelidonine.

Free energy landscape (FEL) analysis is a powerful method for exploring protein folding (Altis et al., 2008). To investigate the energy minima and conformational landscapes of PDGFRβ and its complex systems, we constructed FELs using the first two principal components (PCs) obtained from the PCA analysis (Figure 7). FELs provide a valuable visual representation of the potential energy surface and help identify low-energy states and the most stable conformations of the protein-ligand complexes. By mapping the conformational space using the first two PCs, we gained valuable insights into the potential thermodynamic stability and dominant structural features of the systems. The deeper blue in FELs indicated the lower energy conformations of the protein. As illustrated, the free PDGFRβ, before attaining a global minimum, had multiple local minima surrounded by large basins (Figure 7A). The FEL plots showed that the binding of Genostrychnine and Chelidonine slightly disturbed the size and position of the local and global minima of PDGFRβ (Figures 7B,C), acquiring different states and few to multiple basins in Genostrychnine and Chelidonine, respectively (Figures 7B,C). Overall, the FEL plots suggested that both complexes were stable, attaining the lowest minimum conformation and may not lead to the abnormal unfolding.

### MMPBSA analysis

MMPBSA analysis was carried out to estimate the binding free energy of the PDGFRβ protein-ligand complexes using the gmx_MMPBSA module in GROMACS. Binding energy is a thermodynamic parameter that shows the change in energy associated with the formation of a bond, and it can be used to describe the strength of the interactions between a ligand and a protein. The binding free energy components, including van der Waals force and its average standard deviation complexes obtained from the MMPBSA analysis, are depicted in Table 5. The results indicate that all the PDGFRβ-ligand complexes show promising binding free energies forming stable complexes. Among all the complexes, the PDGFRβ-Genostrychnine was found to have the highest binding affinity, signifying the formation of a more stable complex.

**TABLE 5:** MM-PBSA calculations of binding free energy for PDGFRβ-ligand complexes.

*Complex*	*ΔVDWAALS*	*ΔE*_*EL*_	*ΔE_PB_*	*ΔE*_*NPOLAR*_	*ΔG_GAS_*	*ΔG_SOLV_*	*Standard deviation*	${∆G}_{Total\;\left(kJ/mol\right)}$
PDGFRβ-Genostrychnine	−26.08	−144.18	149.69	−3.28	−170.27	146.41	4.95	−23.85
PDGFRβ-Chelidonine	−21.21	−4.35	19.22	−2.54	−25.56	16.68	2.39	−8.87
PDGFRβ-Sunitinib	−34.83	−0.66	17.93	−3.71	−35.48	14.22	2.62	−21.26

## Discussion

We explored the potential of bioactive phytochemicals as inhibitors of PDGFRβ through a multitier virtual screening approach involving molecular docking, ADMET analysis, PASS analysis, and all-atom MD simulations followed by essential dynamics. The results demonstrate that the identified phytochemical hits have the potential to show significant binding affinities with PDGFRβ and possess favorable ADMET properties, making them promising candidates for further investigation in drug development. Initially, the molecular docking analysis identified the top 30 phytochemical hits with significant binding affinities to PDGFRβ. These compounds showed calculated binding energies of ≥ −9.5 kcal/mol and pKi values of ≥6.9, indicating strong interactions with the receptor. In comparison, the control compounds exhibited binding energies and pKi values of −7.3 kcal/mol (pKi = 5.35) and −9.1 kcal/mol (pKi = 6.67), respectively. The higher binding energies and pKi values of our top 30 phytochemical hits indicate stronger interactions with PDGFRβ than the known inhibitors, Imatinib and Sunitinib, indicating their potential as more effective inhibitors.

The ADMET property analysis exhibited favorable properties for absorption, distribution, metabolism, and excretion for only four phytochemicals and the two control compounds, imatinib and sunitinib. However, the control compounds were found to exhibit toxicity compared to selected phytochemicals. Importantly, these compounds were devoid of any PAINS patterns, indicating that they are not likely to interfere with the assay results during drug screening. The investigation of phytochemicals’ biological properties through PASS analysis provides valuable insights into their potential activities (Lagunin et al., 2000).

The PASS analysis revealed that two compounds, Genostrychnine and Chelidonine, exhibited favorable biological properties as antineoplastic alkaloids, platelet adhesion inhibitors, apoptosis agonists, and phosphatase inhibitors, making them promising candidates against PDGF receptor tyrosine kinases. Their antineoplastic activity suggests they can inhibit cancer cell growth and proliferation, which PDGFRβ often drives. Additionally, their ability to inhibit platelet adhesion, induce apoptosis, and inhibit phosphatase activity highlights their potential to disrupt PDGFRβ-mediated signaling pathways.

Furthermore, the interaction analysis provided detailed insights into how Genostrychnine and Chelidonine interacted with the active site of PDGFRβ. The analysis demonstrated that both compounds formed strong interactions with the Asp826 residue, a crucial region for the functional activity of the kinase. The complementarity of these compounds within the deep binding pocket of PDGFRβ suggests their potential as ATP-competitive inhibitors of the PDGFRβ kinase.

MD simulations are valuable tools for studying the stability and dynamics of protein-ligand complexes over time (Salsbury Jr, 2010; Yousuf et al., 2022). The simulations performed in this study revealed that both PDGFRβ-Genostrychnine and PDGFRβ-Chelidonine complexes exhibited good stability. The RMSD, RMSF, *R*g, and SASA analyses indicated that the binding of Genostrychnine and Chelidonine to PDGFRβ could lead to stable complexes, with minor fluctuations in some regions. The hydrogen bonding analysis also suggested that both complexes had stable intramolecular and intermolecular H-bonds. The PCA and FEL analyses provided further insights into the conformational behavior of the complexes and indicated their potential thermodynamic stability. Finally, the binding free energy estimates carried out with MMPBSA analysis suggested stable complexes for all the PDGFRβ-ligand combinations. Among all, the PDGFRβ-Genostrychnine exhibited the highest binding affinity, surpassing that of the known control, sunitinib, indicating it is the more stable complex.

Overall, the results presented here are compelling and offer valuable information about the potential of the identified phytochemical hits as PDGFRβ inhibitors. The combination of molecular docking, ADMET analysis, PASS analysis, and all-atom MD simulations provides a comprehensive assessment of the identified compounds, allowing us to make informed decisions on which compounds to prioritize for further experimental studies. This study contributes to the field of drug discovery and development and offers potential avenues for designing novel PDGFRβ inhibitors with the identified phytochemicals as starting points.

Nevertheless, the computational tools and databases used in this study have limitations. Molecular docking and virtual screening are based on static models, potentially missing dynamic interactions in biological environments. Online tools like PAINS filter, ADMET profiling, and PASS predictions provide a preliminary assessment of drug-likeness and toxicity but may not account for all possible pharmacokinetic and pharmacodynamic properties. Nonetheless, this study has several limitations that should be considered in future experimental studies. As it relies entirely on computational methods and *in silico* predictions, the compounds would need to be tested *in vitro* and *in vivo* to confirm their efficacy as PDGFRβ inhibitors. Moreover, the study screened only a limited library of about 5800 phytochemical compounds from the IMPPAT database, potentially missing other effective inhibitor candidates. Despite these limitations, the study provides valuable insights and a foundation for further research into potential PDGFRβ inhibitors for cancer therapeutics.

## Conclusion

PDGFRβ is a pivotal player in cancers and other diseases and acts as a promising target for therapeutic development. As per the previous studies, several inhibitors of PDGFRβ have been discovered to date, but more potent and specific inhibitors of PDGFRβ are still necessary. Hence, to find novel inhibitors of PDGFRβ, we undertook a comprehensive approach that involves virtual screening, MD simulation, and essential dynamics. We thoroughly screened a library of phytochemical compounds obtained from the IMPPAT database and identified two lead compounds: Genostrychnine and Chelidonine. These compounds showed efficient binding as well as the structural orientation within the binding pocket of PDGFRβ, making them potential inhibitors of PDGFRβ. Impressively, they exhibited strong binding affinity and a remarkable specificity for the active site as well as ATP binding site residues of PDGFRβ. The stability, as well as the dynamic behavior of complexes and the free state of PDGFRβ, were explored by performing MD simulation. All three systems are quite stable throughout the simulation without any significant fluctuations. Genostrychnine and Chelidonine represent a notable advancement, prompting additional research and tremendous efforts in drug development. Moreover, our findings provide enough evidence to make these compounds promising for the treatment of cancers and other related anomalies, and their discovery plays a crucial role in the exploration of plant drugs from medicinal plants. In summary, the compounds Genostrychnine and Chelidonine promise a new gateway for the therapeutic development of cancers targeting PDGFRβ.

## Data Availability

The original contributions presented in the study are included in the article/Supplementary Material, further inquiries can be directed to the corresponding authors.

## Abbreviations

PDGFRβ, Platelet-derived growth factor receptor beta; RTK, Receptor tyrosine kinase; MD, Molecular dynamics; HMGB1, High mobility group box 1; TKI, Tyrosine kinase inhibitor; CML, Chronic myelogenous leukemia; GIST, Gastrointestinal stromal tumors; ADMET, absorption, distribution, metabolism, elimination, and toxicity; RMSD, Root-mean-square deviation; RMSF, Root Mean Square Fluctuation; Rg, radius of gyration; SASA, Solvent-accessible surface area; PCA, Principal component analysis; FEL, Free energy landscape.
