# Supplementary material for: Identification of potential bioactive phytochemicals for the inhibition of platelet-derived growth factor receptor β: a structure-based approach for cancer therapy
Source: Front Mol Biosci. 2024 Oct 15;11:1492847. doi: 10.3389/fmolb.2024.1492847 (PMC11518818; doi:10.3389/fmolb.2024.1492847)
Supplement: Supplementary file 1 [file Image1.pdf]

Supplementary Data

Running Head: PDGFRβ inhibitors

Identification of Potential Bioactive Phytochemicals for the Inhibition of Platelet-Derived Growth Factor Receptor β: A Structure-based Approach for Cancer Therapy

Insan Habib[1,2,#], Md Nayab Sulaimani[2,#], Deeba Shamim Jairajpuri[3], Afzal Hussain[4], Taj Mohammad[2], Mohamed F. Alajmi[4], Anas Shamsi[5,*], and Md. Imtaiyaz Hassan[2,*]

[1]Department of Parasitology, Faculty of Science, University of South Bohemia, Ceske Budejovice, Czech Republic.

[2]Centre for Interdisciplinary Research in Basic Sciences, Jamia Millia Islamia, Jamia Nagar, New Delhi 110025, INDIA.

[3]Department of Medical Biochemistry, College of Medicine and Medical Sciences, Arabian Gulf University, Manama, Bahrain.

[4]Department of Pharmacognosy, College of Pharmacy, King Saud University, Riyadh, Saudi Arabia.

[5]Centre of Medical and Bio-Allied Health Sciences Research, Ajman University, Ajman P.O. Box 346, United Arab Emirates.

[#]Equal contribution

[*]*Corresponding Author:*
Md. Imtaiyaz Hassan, Ph.D., FRSB, FRSC.

Centre for Interdisciplinary Research in Basic Sciences

Jamia Millia Islamia, Jamia Nagar

New Delhi 110025, INDIA

E-mail: mihassan@jmi.ac.in

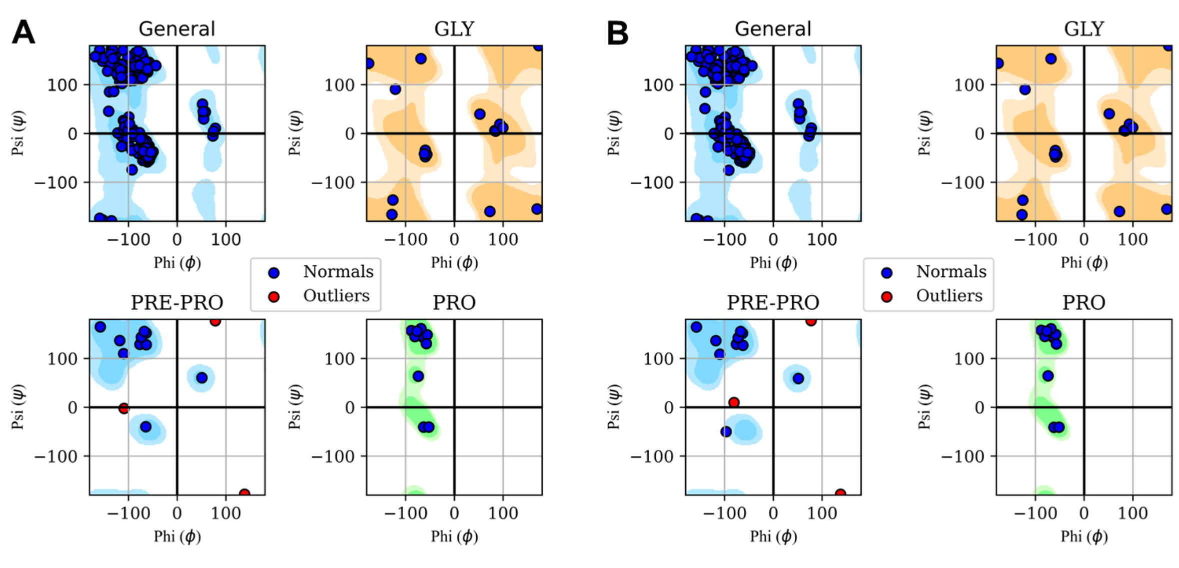

Supplementary Figure S1: Ramachandran plots of PDGFRβ. (**A**) Before energy minimization and (**B**) After minimization of the structure.

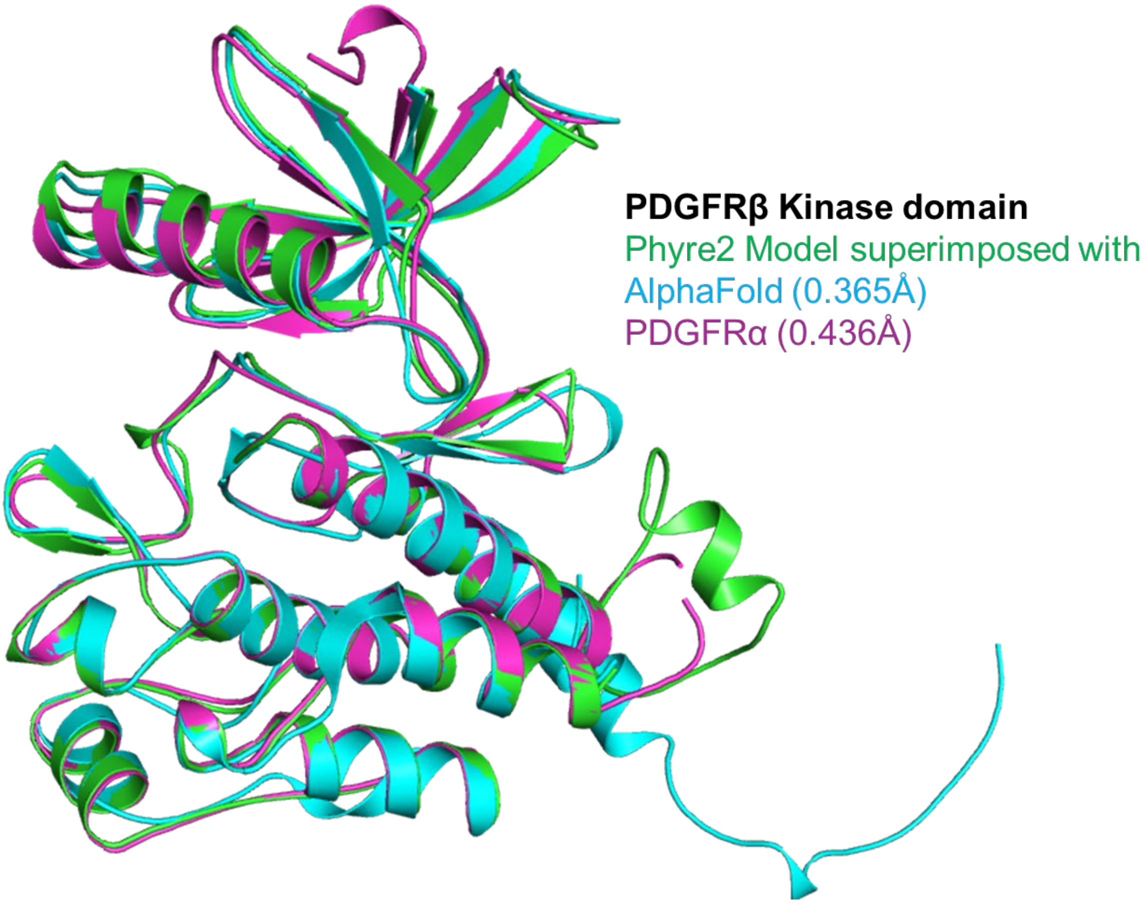

Supplementary Figure S2: The predicted PDGFRβ kinase domain model through Phyre2 (green) and superimposed to AlphaFold (cyan) and PDGFRα (magenta) structure with an RMSD of 0.365Å and 0.436Å, respectively. The figure was drawn in PyMOL.

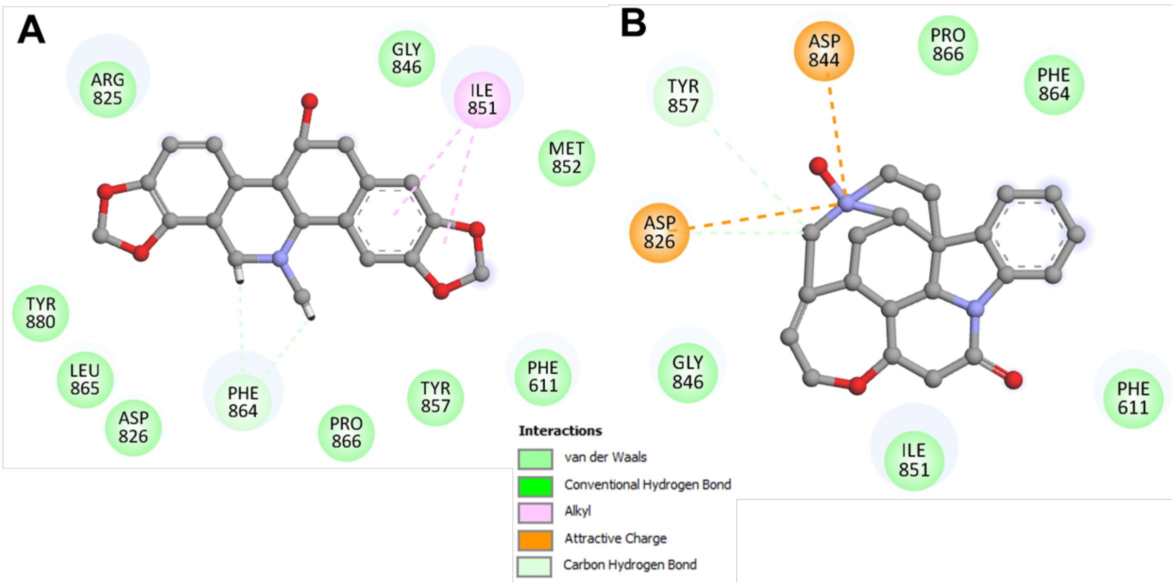

Supplementary Figure S3: Post-molecular dynamics simulations PDGFRβ interactions with (A) Chelidonine and (B) Genostrychnine represented as 2D diagrams. The figure was generated through Discovery Studio Visualizer.
